# Evaluation of a Cubature Kalman Filtering-Based Phase Unwrapping Method for Differential Interferograms with High Noise in Coal Mining Areas

**DOI:** 10.3390/s150716336

**Published:** 2015-07-06

**Authors:** Wanli Liu, Zhengfu Bian, Zhenguo Liu, Qiuzhao Zhang

**Affiliations:** 1 School of Environment Science and Spatial Informatics, China University of Mining and Technology, Xuzhou 221116, China; E-Mails: liuliucumt@126.com (W.L.); winmayliu@hotmail.com (Z.L.); qiuzhaocumt@163.com (Q.Z.); 2 State Key Laboratory for Geomechanics and Deep Underground Engineering, China University of Mining and Technology, Xuzhou 221116, China

**Keywords:** DInSAR, phase unwrapping, Cubature Kalman filter, multi-looks, quality index, fisher distance, minimum cost flow

## Abstract

Differential interferometric synthetic aperture radar has been shown to be effective for monitoring subsidence in coal mining areas. Phase unwrapping can have a dramatic influence on the monitoring result. In this paper, a filtering-based phase unwrapping algorithm in combination with path-following is introduced to unwrap differential interferograms with high noise in mining areas. It can perform simultaneous noise filtering and phase unwrapping so that the pre-filtering steps can be omitted, thus usually retaining more details and improving the detectable deformation. For the method, the nonlinear measurement model of phase unwrapping is processed using a simplified Cubature Kalman filtering, which is an effective and efficient tool used in many nonlinear fields. Three case studies are designed to evaluate the performance of the method. In Case 1, two tests are designed to evaluate the performance of the method under different factors including the number of multi-looks and path-guiding indexes. The result demonstrates that the unwrapped results are sensitive to the number of multi-looks and that the Fisher Distance is the most suitable path-guiding index for our study. Two case studies are then designed to evaluate the feasibility of the proposed phase unwrapping method based on Cubature Kalman filtering. The results indicate that, compared with the popular Minimum Cost Flow method, the Cubature Kalman filtering-based phase unwrapping can achieve promising results without pre-filtering and is an appropriate method for coal mining areas with high noise.

## 1. Introduction

Phase unwrapping is one of the most important processing steps in difference interferometric synthetic aperture radar (DInSAR), which is an effective and appropriate technique that is used for monitoring deformation in large areas [1,2,3]. Numerous phase unwrapping methods have been proposed previously and several detailed studies of these methods have been performed. The methods can be generally divided into two types [4,5]. The first type is based on using the path-following integral to calculate the unwrapped phase, such as branch-cut, region-growing, minimum discontinuity or minimum cost flow (MCF) networks [6,7,8,9]. The basic idea is to isolate and/or mask problematic zones containing residues or discontinuity points by selecting the integral path by different strategies. These methods have the advantages of a quick calculation speed and a precise unwrapped phase in highly coherent areas. The second type of method provides a global solution based on the minimum norms theory. The typical methods are the weighted and the unweighted least-squares phase unwrapping algorithms [10], which obtain solutions by integrating the residues to minimize the gradient differences. Due to the fact that there is some noise in the interferograms, noise filtering is necessary before phase unwrapping (usually called pre-filtering) [11,12,13,14]. However, the pre-filtering step can introduce additional problems. Noise-free areas may be affected by the changed or impaired solution, and the information contained in noisy pixels is usually lost or distorted and cannot be recovered.

From our perspective, the main goal of phase unwrapping is to recover as much information as possible from the interferogram, including the noisy pixels. The filter-based phase unwrapping algorithms have been considered to be appropriate methods [15,16,17,18,19,20,21,22], which transform the phase unwrapping problem into state estimation, simultaneously performing noise filtering and phase unwrapping.

Kim and Griffiths analyzed the potential advantages of Kalman Filtering applied to phase unwrapping [15]. Loffeld and Nies *et al.* presented phase unwrapping methods based on extended Kalman Filtering (EKF) [16,17]. For those algorithms, the next filter state is calculated using only the two adjacent pixels and working along a fixed direction (in the vertical or horizontal direction), which usually leads to a loss in unwrapping accuracy, and potentially even failure because the method travels directly through low quality areas. Osmanoǧlu presented a new EKF approach, which resolves the 2π ambiguity in a computationally efficient way and makes use of the information from all neighbors in the prediction step [18]. A 3-D phase unwrapping method based on EKF was also then investigated to calculate DEM by Osmanoǧlu [20]. For all these EKF based phase unwrapping algorithms, a non-linear observation model performs the linearization approximation for the EKF, which always leads to a loss of high-order phase information, resulting in rapid decreases in the phase unwrapping accuracy in low quality areas. To address this, Xie and Pi presented a new phase unwrapping algorithm based on an unscented Kalman Filter with a path-following strategy and an omni-directional local phase slope estimator [21]. Martinez-Espla *et al.* presented a new phase unwrapping algorithm which combines particle filtering with artificial-intelligence search strategies [19].

It can be observed that the current filter-based phase unwrapping methods not only rely on a linear model or nonlinear model, but also are related to the path-following strategies. It is known that multi-looking is a basic method for speckle suppression of SAR images. One of our research areas is located in Gujiao County, Taiyuan, Shanxi Province, China. Three-quarters of this area feature large mountains with steep slopes, ravines and extensive vegetation, so the differential interferograms usually contain significant noise. Furthermore, repeated excavation taking place in the lower coal seams usually induces bigger and faster deformations. Therefore, the main objective of this study is to find or propose a proper phase unwrapping method which can retain as much detailed information as possible and get the best deformation information.

The Cubature Kalman Filter (CKF) is considered to be an effective, simple and efficient tool in many nonlinear fields [23,24,25,26]. In this paper, a CKF-based phase unwrapping (CKFPU) method is firstly presented. Cubature points are only used in the measurement process and not in the prediction step, so the method is suitable for unwrapping models that use linear prediction equations but nonlinear measurement equations. This strategy not only reduces the calculation burden but can also avoid underestimation of the error covariance caused by linearization of the nonlinear measurement model. Two factors related to the CKFPU method—the number of multi-looks and the path-guiding indexes—are then analyzed and tested under different conditions. Additionally, the performance of the proposed method is compared with the conventional MCF unwrapping method, which is currently considered the most appropriate algorithm in rural areas. The results indicate that the proposed method is an effective and feasible strategy for DInSAR phase unwrapping in high noise areas and can avoid the difficulties introduced by pre-filtering.

This paper is organized as follows: firstly, phase unwrapping theory and local phase slope estimation are introduced in Section 2. Section 3 further elaborates the system model, algorithms and the key factors on CKFPU. Tests and analysis are given in Section 4. The conclusion is given in the final section.

## 2. Phase Unwrapping Theory and Local Phase Slope Estimation

### 2.1. Phase Unwrapping Theory

For a complex SAR interferogram, the polar notation at pixel *k* can be expressed as [16,21]:

(1) $$z(k)=a(k)\exp(j\tilde{\varphi }(k))$$

where *z*(*k*) is the complex interferometric measurement; *a*(*k*) is the observed interferometric amplitude and $\tilde{\varphi }(k)$ is the modulo 2π mapped interferometric phase, also called the wrapped phase, which has the following mathematical expression:

(2) $$\tilde{\varphi }(k)={\left[\varphi (k)+\varepsilon_{\varphi }(k)\right]}_{|2\pi }={\left[\varphi (k)+{\left[\varepsilon_{\varphi }(k)\right]}_{|2\pi }\right]}_{|2\pi }={\left[\varphi (k)+{\tilde{\varepsilon }}_{\varphi }(k)\right]}_{|2\pi }=\varphi (k)+{\tilde{\varepsilon }}_{\varphi }(k)\pm 2n\pi \in (-\pi ,\pi ]$$

where *ϕ*(*k*) is the true unambiguous phase, *ε_ϕ_*(*k*) refers to the true phase error and ${\tilde{\varepsilon }}_{\varphi }(k)$ is the mapped phase error.

It is essential to obtain the phase difference between consecutive pixels to obtain the unwrapped phase. It is calculated as:

(3) $${\tilde{\Delta }}_{\varphi }(k)={\left[\tilde{\varphi }(k+1)-\tilde{\varphi }(k)\right]}_{|2\pi }$$

Using Equation (2) and the properties of the modulus operation, Equation (3) can be rewritten as:

(4) $${\tilde{\Delta }}_{\varphi }(k)={\left[\delta_{\varphi }(k)+{\left[{\tilde{\varepsilon }}_{\varphi }(k+1)-\varepsilon_{\varphi }(k)\right]}_{|2\pi }\right]}_{|2\pi }$$

where $\delta_{\varphi }(k)$ is the true discrete phase derivative (or phase slope or phase gradient, $\delta_{\varphi }(k)=\varphi (k+1)-\varphi (k)$) whose modulus should always be less than π. It can be seen from Equation (4) that, if there are no phase errors, *i.e.*, ${\tilde{\Delta }}_{\varphi }(k)=\delta_{\varphi }(k)$, the unwrapped phase *ϕ* can be obtained by the following recursive expression:

(5) $$\varphi (k+1)=\varphi (k)+{\tilde{\Delta }}_{\varphi }(k)$$

However, phase errors always occur in normal interferograms so the phase slope calculated using Equation (3) is biased in most cases and the estimated phase based on Equation (5) is not reliable, especially in high noise areas. According to previous studies [16,19,21,22], the local frequency of complex interferogram provides a common solution to obtain the local phase slope estimation.

### 2.2. Phase Slope Estimation

InSAR signals are usually considered to be stable in a small local area. Accordingly, a complex sinusoidal signal in the local window (*B_m_* *× B_n_*) can be generally expressed as [21,27,28]:

(6) $$z(m,n)=a(m,n)\exp(j\tilde{\varphi }(m,n))=a(m,n)\exp[j2\pi (f_{x}n+f_{y}m)]+w(m,n)$$

where *m* = 1,2,…,*B_m_*, *n* = 1,2,…,*B_n_*; *m* and *n* refer to the row and column indices respectively; *B_m_* and *B_n_* are the row and column length of the local window respectively; *a*(*m*,*n*) is the random amplitude of the complex signal; *f_x_* and *f_y_* refer to the true local frequency in the row and column direction respectively; and *w*(*m*,*n*) is the corresponding noise.

The local frequency in the local window is estimated by a maximum likelihood estimator in this paper. The Cramer-Rao bound for local frequency estimation error is as follows:

(7) $$\begin{array}{l} c(f_{x})=\frac{1-r^{2}}{r^{2}B_{m}B_{n}(B_{m}^{2}-1)}\\ c(f_{y})=\frac{1-r^{2}}{r^{2}B_{m}B_{n}(B_{n}^{2}-1)} \end{array}$$

where *r* is the coherence coefficient for the local area of the interferogram. The phase gradient estimation value from pixel (*a*,*s*) to pixel (*m*,*n*) in a small area can be calculated by the local frequency estimator:

(8) $${\tilde{\delta }}_{\varphi }=2\pi {\vec{f}}_{x(a,s)}(m-a)+2\pi {\vec{f}}_{y(a,s)}(n-s)$$

where ${\vec{f}}_{x(a,s)}$ and ${\vec{f}}_{y(a,s)}$ are the frequency estimation values in the local window around the pixel (*a*,*s*). From Formulas (7) and (8), the estimation error variance for the local phase gradient $\sigma_{w}^{2}$ can be calculated by:

(9) $$\sigma_{w}^{2}={\left(2\pi \right)}^{2}[{\left(m-a\right)}^{2}c(f_{x})+{\left(n-s\right)}^{2}c(f_{y})]$$

From above, it can be seen that it is almost impossible to obtain the true phase slope from the interferograms directly. Instead, its statistic model can be calculated by using estimators, which will be used for the filter-based unwrapping method that will be described in the following sections.

## 3. Cubature Kalman Filtering Based Phase Unwrapping

### 3.1. System Model for Phase Unwrapping

#### 3.1.1. State-Space Equation

A simple but effective state-space model for the required unambiguous phase has been described previously [16,17,18,21] as:

(10) $$\begin{array}{l} x(k)=\varphi (k)\\ x(k+1)=x(k)+\delta_{\varphi }(k)\\ \delta_{\varphi }(k)={\vec{\delta }}_{\varphi }(k)+w(k) \end{array}$$

where ${\vec{\delta }}_{\varphi }(k)$ and *w*(*k*) are the phase slope estimation and its corresponding estimation error:

$$E[w(k)]=0,\;Q(k)=E[w(k)w{\left(j\right)}^{T}]=\sigma_{w}^{2}\delta (k,j),\;\delta (k,j)=\left\{ \begin{array}{l} 1,k=j\\ 0,k\neq j \end{array}\right.$$

where *Q*(*k*) is the covariance matrix of the phase slope estimation error and $\sigma_{w}^{2}(k)$ is the phase slope estimation error variance at pixel (*k*).

#### 3.1.2. Observation Equation

The observation model at pixel *k* is usually expressed as follows [16,17,18,21]:

(11) y(k)=[Im{z(k)}a(k)Re{z(k)}a(k)]=[sin(φ(k))cos(φ(k))]+[v1(k)v2(k)]=h[φ(k)]+v(k)

where *v*_1_(*k*) and *v*_2_(*k*) are the errors in the real and imaginary parts of the complex measurements, respectively. In a strict sense, the right-hand side of the second equal sign is actually more of a definition or a model than an equality [16], but it is written as shown for simplicity:

$$E[v(k)]=0,\;R(k)=E[v(k)v{\left(j\right)}^{T}]=diag(\sigma_{v}^{2}(k)\delta (k,j)),\;\sigma_{v}^{2}(k)=\frac{1}{S_{SNR}^{k}}$$

where $S_{SNR}^{k}$ is the signal to noise ratio of pixel *k*.

Thus, the state equation and the non-linear measurement equation can be written as:

(12) $$x(k+1)=x(k)+{\vec{\delta }}_{\varphi }(k)+w(k)$$

(13) $$y(k+1)=h[x(k+1)]+v(k+1)=\left[\begin{array}{l} \sin(x(k+1))\\ \cos(x(k+1)) \end{array}\right]+\left[\begin{array}{l} v_{1}(k+1)\\ v_{2}(k+1) \end{array}\right]$$

### 3.2. CKF Phase Unwrapping (CKFPU) Algorithms

#### 3.2.1. One-Dimensional CKFPU Algorithm

For the state and measurement models in Section 3.1.1 and Section 3.1.2, the CKF and state estimation can be completed as follows: assume that the state vector and its corresponding estimation error covariance matrix for pixel *k* are $\vec{x}(k)$ and $P_{xx}^{+}(k)$ respectively. Then, the one-step prediction value ${\vec{x}}^{-}(k+1)$ and its prediction error covariance matrix $P_{xx}^{-}(k+1)$ can be calculated as:

(14) $${\vec{x}}^{-}(k+1)=\vec{x}(k)+{\vec{\delta }}_{\varphi }(k)+w(k)$$

(15) $$P_{xx}^{-}(k+1)=P_{xx}^{+}(k)+Q(k)$$

Since the state model of phase unwrapping is a linear model, a simplified CKF method is proposed. In the simplified CKF method, the linear Kalman Filtering prediction step is used to reduce computation, and a CKF measurement step is then implemented to handle the nonlinear measurement model. The measurement update step of CKF for phase unwrapping is as follows: 

Calculate the cubature points and the weights based on the spherical-radial cubature rule:

$\xi_{i}=\sqrt{\frac{m}{2}}{\left[1\right]}_{i}$, $\omega_{i}=\frac{1}{m}$, where *i* = 1,2,…,*m*, *m* = 2*n_x_* and *n_x_* is the dimension of the state vector.

Factorize the error covariance:

(16) $$P_{xx}^{-}(k+1)=S_{xx}(k+1)+S_{xx}^{T}(k+1)$$

where *S_xx_* can be obtained by Cholesky decomposition or singular value decomposition.

Calculate the cubature points and weights:

(17) $$X_{i,k+1}=S_{xx}(k+1)\xi_{i}+{\vec{x}}^{-}(k+1)$$

Evaluate the propagated cubature points:

(18) $$Z_{i,k+1}=h(X_{i,k+1},v_{k+1})$$

where *v_k_* is the measurement noise, which has a Gaussian distribution.

The predicted measurement ${\vec{y}}^{-}(k+1)$ and its corresponding error covariance matrix $P_{yy}^{-}(k+1)$ for pixel (*k* + 1) can be calculated based on the observation equation as:

(19) $$\left\{ \begin{array}{l} {\vec{y}}^{-}(k+1)=\sum_{i=1}^{m}\omega_{i}Z_{i,k+1}\\ P_{yy}^{-}(k+1)=\sum_{i=1}^{m}\omega_{i}Z_{i,k+1}Z_{i,k+1}^{T}-{\vec{y}}^{-}(k+1){\left({\vec{y}}^{-}(k+1)\right)}^{T}+R(k+1) \end{array}\right.$$

The cross-covariance matrix $P_{xy}^{-}(k+1)$ of the predicted measurements can be calculated as:

(20) $$P_{xy}^{-}(k+1)=\sum_{i=1}^{m}\omega_{i}X_{i,k+1}Z_{i,k+1}^{T}-{\vec{x}}^{-}(k+1){\left({\vec{y}}^{-}(k+1)\right)}^{T}$$

Finally, the state estimation $\vec{x}(k+1)$ and its covariance matrix $P_{xx}^{+}(k+1)$ for pixel (*k* + 1) are as follows:

(21) $$\left\{ \begin{array}{l} K(k+1)=P_{xy}^{-}(k+1)/P_{yy}^{-}(k+1)\\ \vec{x}(k+1)={\vec{x}}^{-}(k+1)+K(k+1)(y(k+1)-({\vec{y}}^{-}(k+1))\\ P_{xx}^{+}(k+1)=P_{xx}^{-}(k+1)-K(k+1)P_{xy}^{-}(k+1)K^{T}(k+1) \end{array}\right.$$

where *K*(*k* + 1) is the Cubature Kalman gain matrix. Based on the above steps, a one-dimensional phase unwrapping model based on CKF is developed. It can be observed that the CKF algorithm performs noise filtering and phase unwrapping at the same time by fusing the information extracted from the complex interferogram.

#### 3.2.2. Two-Dimensional CKFPU Algorithm

**Figure 1:**
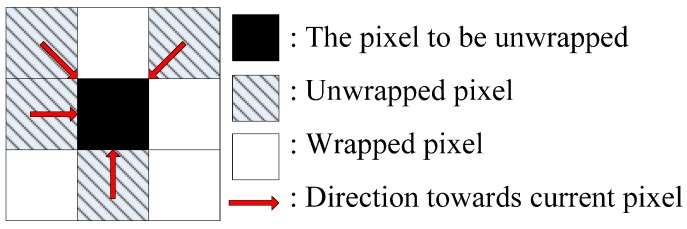
Prediction principle of two-dimensional CKFPU.

The CKFPU is combined with a path-following strategy and omnidirectional local phase slope estimation, to guide the CKFPU by the phase quality so that it operates from high-quality regions to low-quality regions. The prediction estimation of any pixel will be calculated by weighting the corresponding unwrapped adjacent pixels, as shown in Figure 1. Hence, for the two-dimensional CKFPU algorithm, the prediction equation can be modified as follows:

(22) $${\vec{x}}_{m,n}^{-}=\sum_{\left(a,s)\in (B,L\right)}d(a,s){\vec{x}}_{\left[(m,n)|\left(a,s\right)\right]}$$

(23) $$P_{m,n}^{-}=\sum_{\left(a,s)\in (B,L\right)}d(a,s)\left(P_{\left[(m,n)|\left(a,s\right)\right]}+Q_{\left(a,s\right)}\right)$$

where ${\vec{x}}_{m,n}^{-}$ is the predicted state vector of pixel (*m*,*n*) and $P_{m,n}^{-}$ is its corresponding error matrix; ${\vec{x}}_{\left[(m,n)|\left(a,s\right)\right]}$ denotes the estimated state vector of the adjacent unwrapped pixels and $P_{\left[(m,n)|\left(a,s\right)\right]}$ is the corresponding error matrix; *Q*_(*a*,*s*)_ is the estimated error variance matrix for phase slope at pixel (*a*,*s*); *B* denotes eight adjacent pixels for pixel (*m*,*n*) and *L* denotes the whole image. The optimal weight *d*(*a*,*s*) can be calculated as follows [21]:

(24) d(a,s)=[P(a,s)×1SNR(a,s)]−1g(a,s)∑(a,s)∈(B,L)([P(a,s)×1SNR(a,s)]−1g(a,s))

(25) $$g(a,s)=\left\{ \begin{array}{l} 1,(a,s)pixel-unwrapped\\ 0,(a,s)pixel-wrapped \end{array}\right.$$

Equation (24) indicates that contiguous unwrapped pixels with a greater estimation error covariance make a smaller contribution to the current pixel.

### 3.3. The Main Factors Affecting CKFPU Performance

Since multi-looking is a basic method for speckle suppression of SAR images, it can not only filter out noise, but also reduce the dimensionality of SAR data. However, multi-looking may also filter out relevant details, thus affecting the performance of phase unwrapping. As the path-following strategy is used in CKFPU method, several path-guiding indexes are tested.

#### 3.3.1. Number of Multi-Looks

A previous study [29] investigated the relationship between the maximum/minimum detectable deformation gradient, the number of multi-looks and the coherence, and established empirical function models of the maximum/minimum detectable deformation gradients when the number of multi-looks is 1, 5 and 20. It was observed that the greater the number of multi-looks, the smaller the detectable maximum deformation gradient. However, different unwrapping methods will lead to different unwrapped results even with the same maximum/minimum detectable deformation gradients. This paper compares the performance of the CKFPU method where the number of multi-looks is 1, 2 and 4.

#### 3.3.2. Quality Indexes for Guiding Path Tracking

The state-space model (prediction step) of the CKFPU indicates that the prediction of the next step is based on a given path. The path is usually selected based on certain quality indexes. Six types of quality indexes were described and compared in the reference [30]. The results indicate that the Fisher Distance (FD) has the best performance in most cases. However, the study noted that different results might be obtained with different unwrapping functions. Taking into consideration the high noise and fast deformation characteristic of our research area, three typical unwrapping paths—Maximum Coherence (MC), Phase Derivative Variance (PVD) and FD—are used to guide CKFPU from the high-quality regions to the low-quality regions.

#### Maximum Coherence

Coherence can be used as a quality map to signify the coherence degree for each area of the SAR interferogram. Generally, the higher the coherence is, the better the pixel quality is. Therefore, the coherence map is regarded as the most direct and common way to measure the interferogram quality. In this paper, the coherence map is from the GAMMA software processing.

#### Phase Derivative Variance

PDV is one of the earliest methods that was developed to define the unwrapping path and is calculated over the four-cell neighborhood and the pixel itself (five pixels in total). For two-dimensional data, the PDVs are summed over both the *x* and *y* dimensions. The PDV of pixel *k* can be calculated as:

(26) $$PDV_{k}={\sum \left(\Delta_{i,j}^{x}-{\bar{\Delta }}_{m,n}^{x}\right)}^{2}+{\sum \left(\Delta_{i,j}^{y}-{\bar{\Delta }}_{m,n}^{y}\right)}^{2}$$

where $\Delta_{i,j}^{x}={\left[\Psi_{i+1,j}-\Psi_{i,j}\right]}_{|2\pi }$, $\Delta_{i,j}^{y}={\left[\Psi_{i,j+1}-\Psi_{i,j}\right]}_{|2\pi }$, $\Psi_{i,j}$ is the wrapped phase of the interferograms, *N* is the size of the calculated window, and ${\bar{\Delta }}_{m,n}^{x}$ and ${\bar{\Delta }}_{m,n}^{y}$ are the mean of $\Delta_{i,j}^{x}$ and $\Delta_{i,j}^{y}$ respectively. From the formula above, it is found that the PVD shows spatial similarity with the interferometric phase. A smaller PDV represents a better quality.

#### Fisher Distance

FD is a measure of phase similarity based on Fisher information theory [20], expressed as:

(27) $$FD=\frac{1}{4N}\sum_{n=1}^{N}\left(\frac{{∠}^{2}(\varphi_{n}\varphi_{0}^{*})(\sigma_{\varphi 0}^{2}+\sigma_{\varphi n}^{2})}{\sigma_{\varphi 0}^{2}\sigma_{\varphi n}^{2}}+\log(4\pi^{2}\sigma_{\varphi 0}^{2}\sigma_{\varphi n}^{2})\right)$$

where *FD* is the FD for the current pixel calculated over its neighborhood; *N* is the number of neighboring pixels; $∠(\varphi_{n}\varphi_{0}^{*})$ is the complex difference of the phase angles, and $\sigma_{\varphi 0}$ is the expected standard deviation of the phase values calculated from coherence [31]. From Equation (27), it is easily seen that the FD quality index is a combination of the phase derivative and the expected phase variance (based on coherence). Compared with MC and PDV, the FD index is more robust and comprehensive for guiding the path of phase unwrapping, as it restrains both the spatial diversity and coherence randomness. A smaller value of FD usually indicates a better quality.

## 4. Experiments

### 4.1. Dataset Introduction and DInSAR Processing Strategy

#### 4.1.1. Dataset Introduction

Three datasets were used to measure the effects of the proposed CKFPU method which are all TerraSAR-X datasets, from the relatively high-spatial resolution and short-time repeat cycle SAR satellite TerraSAR-X launched by Germany on 15 June 2007. Its basic parameter values are listed in Table 1.

The target areas of these three datasets are all located in a coal mining working face. Table 2 shows the specifications of the interferometry pairs.

**Figure 2:**
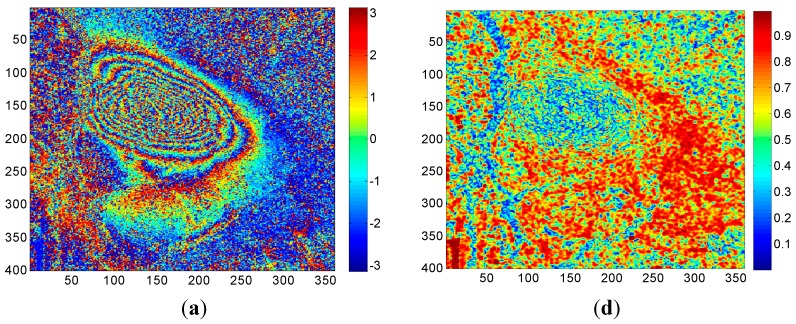

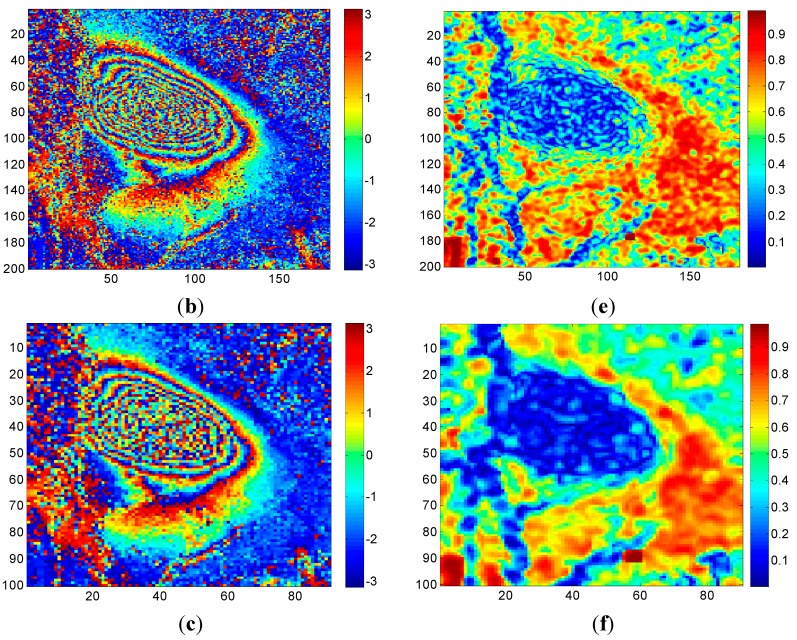
Interferograms (left) and coherence maps (right) under different numbers of multi-looks in the working face 18a203: (**a**) interferogram of 1 × 1 look; (**b**) interferogram of 2 × 2 looks; (**c**) interferogram of 4 × 4 looks; (**d**) coherence map of 1 × 1 look; (**e**) coherence map of 2 × 2 looks; (**f**) coherence map of 4 × 4 looks.

Dataset A: The data was acquired on 4 January 2013 and 15 January 2013 covering the 18a203 working face of the Tunlan coal mine, which is located in the Gujiao mining area of Taiyuan. The working face 18a203 was active during 4 January 2013 and 15 January 2013 by collecting and analyzing the corresponding coal mining archive and GPS measurement data. There was about 20–30 cm (80–100 radians in the line of sight direction) of subsidence during this repeated cycle. From the SAR interferograms and coherence maps (Figure 2), it is clear that there is fast subsidence with severe decorrelation in the center of the working face and it has high noise.

**Table 1:** TerraSAR-X Parameters used in this study.

Parameters	Values
Frequency	9.6 GHZ
Wavelength	3.1 cm
Polarisation	HH
Swath Width	50 km
Incidence Angle	~26°
Range Pixel Spacing	0.9 m
Azimuth Pixel Spacing	1.9 m
Orbit Repeat Cycle	11 days
Precise Orbit Accuracy	~10 cm

**Table 2:** Specifications of interferometry combination for DInSAR.

Datasets	Target Working Face	Master	Slave	Perpendicular Baseline B_⊥_/m	Temporal Baseline B_T_/day
A	18a203	4 January 2013	15 January 2013	−103.3	11
B	52304	2 April 2013	24 April 2013	−107.2	22
C	18a203	16 September 2012	27 September 2012	−3	11
		27 September 2012	8 October 2012	−7.2	11
		8 October 2012	19 October 2012	−70.2	11
		19 October 2012	10 November 2012	−30.5	22
		10 November 2012	21 November 2012	62.2	11
		21 November 2012	2 December 2012	160.7	11
		2 December 2012	13 December 2012	−36.3	11
		13 December 2012	24 December 2012	−99.8	11

Dataset B: The data was acquired on 2 April 2013 and 24 April 2013 covering the 52304 working face of the Daliuta coal mine, which is located in the Shendong mining area. This area features flat ground or gently rolling slopes, and is mainly barren grassland or sandstone. During this repeated cycle, the working face was in the final period of mining and appeared to be slowly sinking.

Dataset C: The data was acquired from 16 September 2012 to 15 January 2013 covering the 18a203 working face of the Tunlan coal mine. Due to vegetation, dew and snow, some interferograms have a high noise element.

#### 4.1.2. DInSAR Processing Strategy

For DInSAR in this paper, a two-pass method was used and the relief DEM was generated by ourselves with 4 m resolution, and selected as the external DEM. The pre-processing was implemented by GAMMA software to obtain the SAR interferogram and coherence map. The phase unwrapping step was completed by our own software.

### 4.2. Results and Analysis

#### 4.2.1. Case 1

Due to the large phase gradient, some deformation signals could not be derived correctly by phase unwrapping. However, different schemes or parameter settings for the same method often leads to different unwrapped results. Therefore, we require that an appropriate method is found that has better unwrapping performance on the CKF PU method.

Two tests were designed to evaluate the unwrapping effect including the number of multi-looks and quality index for path-guiding. While each index was tested, the other indexes remained constant. The detailed schemes for these tests can be listed as follows:

Test 1: Test on the effect of the number of multi-looks on the CKFPU method.

Scheme 1: FD was used as the path-guiding quality index, the single-looked differential interferogram was the wrapped objective.

Scheme 2: FD was used as the path-guiding quality index, the two-looked differential interferogram was the wrapped objective.

Scheme 3: FD was used as the path-guiding quality index, the four-looked differential interferogram was the wrapped objective.

The interferograms and coherence maps are shown in Figure 2. It can be seen that as the number of multi-looks increases, there is a corresponding decrease in the speckle noise. However, some details were also smoothed out, which is unsatisfactory. From Figure 2, we can observe that the single-looked interferogram shows more noise, fringes and detailed information. As the number of multi-looks increases, the spatial-resolution decreases, some detailed information is smoothed out and fringes become vague, which may introduce another problem—the number of fringe decreases and the maximum unwrapping ability will be weakened especially in areas with fast subsidence.

**Figure 3:**
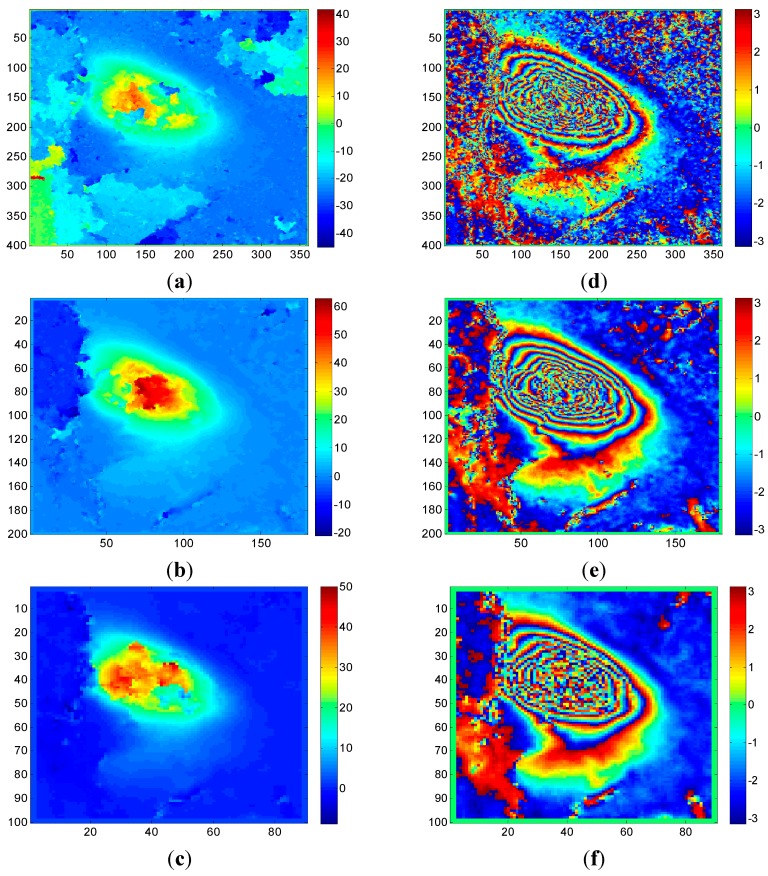
Unwrapped maps (left) and rewrapped maps (right) under different numbers of multi-looks: (**a**) unwrapped map of 1 × 1 look; (**b**) unwrapped map of 2 × 2 looks; (**c**) unwrapped map of 4 × 4 looks; (**d**) rewrapped map of 1 × 1 look; (**e**) rewrapped map of 2 × 2 looks; (**f**) rewrapped map of 4 × 4 looks.

Figure 3 shows the unwrapped results obtained by the CKFPU algorithm. Figure 3a–c are unwrapped maps and Figure 3d–f are the corresponding rewrapped maps. Compared to the interferograms in Figure 2, all of the rewrapped maps possess a distinct characteristic—noise was filtered out. However, the unwrapped results appear quite different from each other. For single-looking, there are several subsidence and upraised areas which are not continuous and do not reflect the actual situation of the study area. This is because the single-looked data includes high noise which plays a key role in these areas. For two-looking, the unwrapped map seems more continuous, since a higher Signal to Noise Ratio of the interferogram can be obtained by two-looking processing. Additionally, compared to single-looking and four-looking results, two-looking obtained the highest subsidence value and had the best unwrapping performance. Based on the collected coal mining logs, this working face was active during the time TerraSAR-X satellite visited the target area and the maximum subsidence of the active working face could reach up to 20–30 cm (80–100 radian in line of sight direction). However, the maximum subsidence achieved by some conventional unwrapping methods and software are all far less than the actual subsidence value. Here, we introduced a qualitative evaluation scheme to estimate the performance of these schemes, which was that the greater the maximum unwrapped phase, the better result, which is closer to the reality. For real data, the rewrapped map and original interferogram are generally compared to evaluate the performance of phase unwrapping methods. The rewrapped map with clearer fringes, less noise and more detailed information is considered to be the better one. From Figure 3, it is clear that Scheme 2 is the best one. For Scheme 3, although the unwrapped map is relatively smoother than the other two, the maximum subsidence is less and the fringes are vaguer. This is because spatial resolution declines as the number of multi-looks increases. In summary, the two-looking method is the most appropriate selection for the given interferogram. It should be noted that how to select a proper number of multi-looks for specific data is not the emphasis in this paper. The main aim of this test is to prove that the number of multi-looks is important to the CKFPU method in study areas with high noise. Additionally, it is also verified that the new method can unwrap the interferograms while filtering out noise.

Test 2 was designed to evaluate the effect of different path-guiding indexes on the CKFPU method, using the same data as used as Test1. Scheme 2 of this test is the same scheme used in Test 1.

Scheme 2: FD was used as the path-guiding quality index; the wrapped objective was the two-looked differential interferogram.

Scheme 4: MC was used as the path-guiding quality index; the wrapped objective was the two-looked differential interferogram.

Scheme 5: PDV was used as the path-guiding quality index; the wrapped objective was the two-looked differential interferogram.

**Figure 4:**
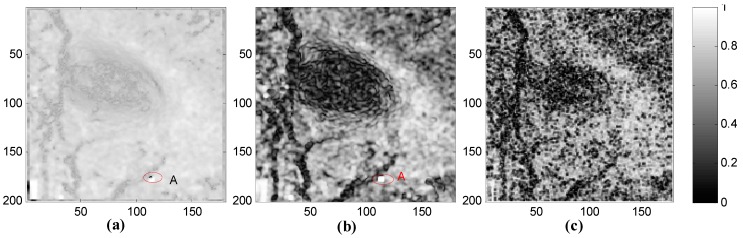
Three phase quality maps with FD, MC and PDV: (**a**) FD quality map; (**b**) MC quality map; (**c**) PDV quality map.

Figure 4 shows the three phase quality maps with each of the different quality indexes. Figure 4a–c are the quality maps calculated by FD, MC and PDV, respectively. A darker color in these graphs represents worse quality, and *vice versa*. At first glance, there are more similarities overall between Figure 4a,b, but there are also obvious differences. On the whole, Figure 4a is brighter than Figure 4b. This is because the values calculated by FD are mainly distributed in the lower range while the values of MC have a better dispersion. In addition, some pixels show low quality in Figure 4a but high quality in Figure 4b. Taking a typical pixel as an example, pixel A is an artificial Corner Reflector and is homologous in the subgraphs. However, pixel A exhibits quite different qualities in each of the three graphs. In Figure 4a, pixel A is very dark which means that the pixel is low quality. This is because FD reflects similarities between each pixel and its surroundings. In this example, the surrounding area of the Corner Reflector is covered by ground objects which are seriously uncorrelated, so the similarity is very low and the FD value is much higher. However, in Figure 4b, the opposite effect is shown. Since MC is used to indicate the correlation between each pixel in the two SLC images, more stable ground objects always lead to a greater coherence. Additionally, there is a slower change from a poor quality area to high quality area in Figure 4c. In other words, it is insensitive to different objects. Equation (29) can be interpreted as meaning that PDV only reflects the statistical characteristics of pixels in a given window without emphasizing the information of the current pixel, so some details are smoothed out. In summary, FD and MC are more sensitive than PDV. Compared with MC, the FD method considers both spatial diversity and coherence randomness, and is more robust and comprehensive for guiding the path of phase unwrapping.

**Figure 5:**
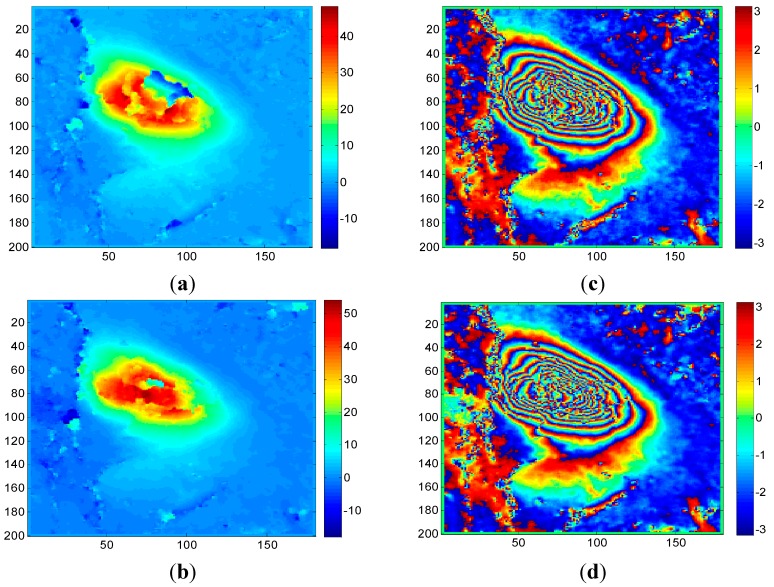
Unwrapped maps (left) and rewrapped maps (right) based on MC and PDV: (**a**) unwrapped map of MC; (**b**) unwrapped map of PDV; (**c**) rewrapped map of MC; (**d**) rewrapped map of PDV.

Figure 5 shows the corresponding unwrapped and rewrapped results guided by MC and PDV. This can be compared with Figure 3b,e, which show the unwrapped and rewrapped results where path tracking is guided by FD. If we focus on the unwrapped maps, it can be seen that the FD method (shown in Figure 3) obtains a more continuous unwrapped phase and a greater maximum subsidence. This fits the subsidence map and interferogram better—the area with dense fringes has large subsidence. This is because the FD index combines the phase derivative with the expected phase variance (based on coherence), which is more robust and suitable for mountainous areas with less persistent scatters. From the rewrapped maps, the MC method retains most of the details, due to the fact that MC only accounts for the correlation without considering the spatial similarity of the differential interferogram. The highly correlated pixels are usually permanent scatters with little noise, so working along this index may lead to a clearer rewrapped map. However, it is not reliable in areas with fast subsidence that are covered in vegetation. For example, the Corner Reflector pixel located in a fast subsidence area is usually treated as high quality pixel by the MC method, due to the high coherence value. This is clearly not reasonable, since fast subsidence can lead to signal aliasing, preventing the real situation of the subsidence from being reflected. If it is used as a reference in earlier steps, there will be more error propagation. The PDV method takes the spatial difference into account without considering the coherence, which may weaken the overall reliability. Therefore, a continuous but low maximum unwrapped phase result is obtained by PDV. In summary, the FD method is more suitable for guiding the path of CKFPU algorithm where there is high noise and large phase gradients.

#### 4.2.2. Case 2

Two datasets (Dataset A and Dataset B) were used to measure the performance of CKFPU in high noise areas. Additionally, the test was also designed to compare the proposed CKFPU algorithm with the MCF method. It should be noted that FD was also used in the MCF method. The pre-filtering steps for the MCF method was conducted using GAMMA software with adaptive filtering and the thresholds were set at 0.1 and a default value of 0.25, respectively. The CKFPU method employed the same settings as Scheme 2 in Case 1.

**Figure 6:**
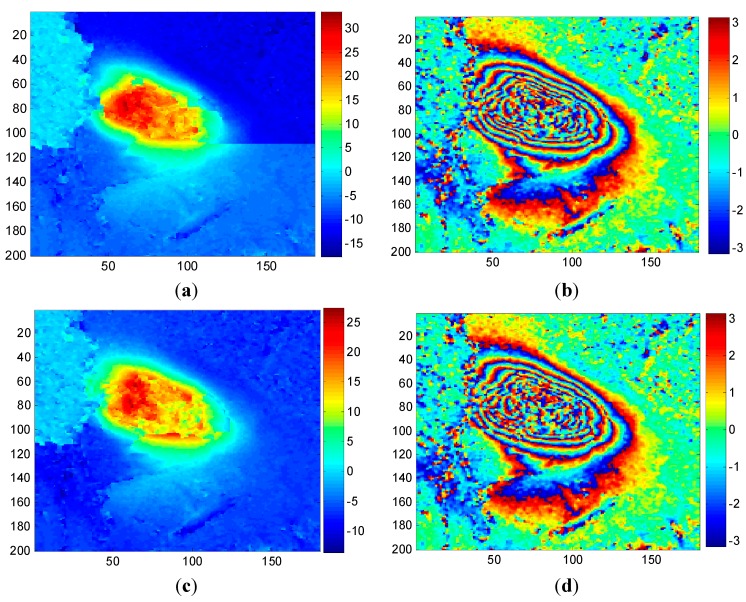
Results based on MCF method: (**a**) unwrapped map with pre-filtering filter threshold set at 0.1; (**b**) rewrapped map of (**a**); (**c**) unwrapped map with adaptive filter threshold set at 0.25; (**d**) rewrapped map of (**c**).

For Dataset A, the same 2 × 2 multi-look interferograms and coherence maps were used for both CKFPU and MCF. By way of explanation, the interferograms and coherence maps are shown in Figure 2b,e. The corresponding results of CKFPU are shown in Figure 3b,e. Figure 6 shows the results of the MCF method only. Figure 6a,b are the unwrapped and rewrapped maps when the pre-filtering threshold is set at 0.1. Figure 6c,d are the unwrapped and rewrapped maps when the pre-filtering threshold is set at the default value 0.25.

**Figure 7:**
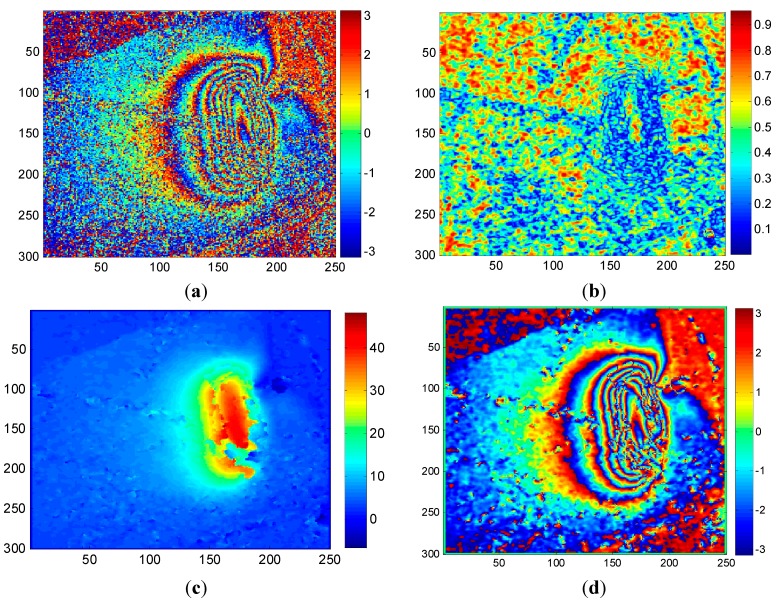

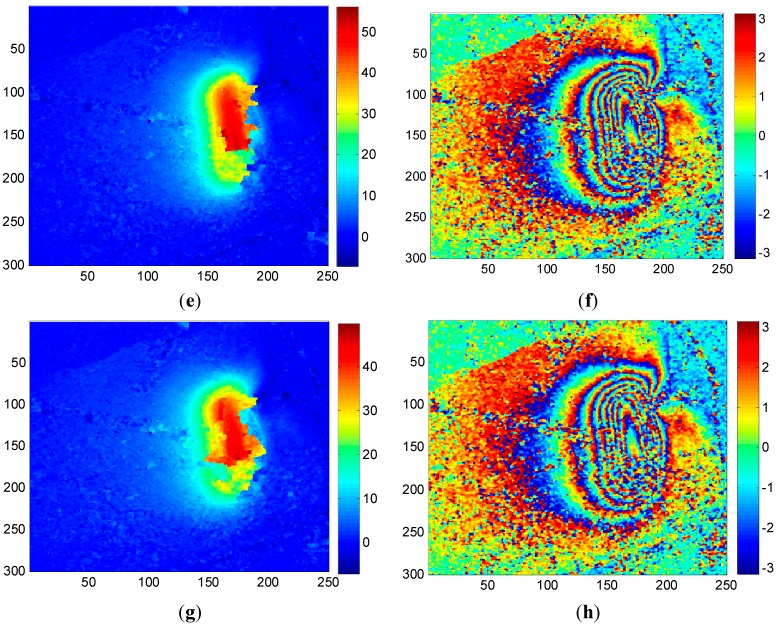
Experimental results for Dataset 2: (**a**) original differential interferogram; (**b**) corresponding coherence map; (**c**) unwrapped map of CKFPU; (**d**) rewrapped map of CKFPU; (**e**) unwrapped map of MCF when the pre-filtering threshold is set at 0.1; (**f**) rewrapped map of (e); (**g**) unwrapped map of MCF based on MCF when the pre-filtering threshold is set at 0.25 and (**h**) its rewrapped map.

Figure 7 shows the differential interferogram, coherence map and the experimental results for Dataset 2. The interferogram and coherence map are shown in Figure 7a,b. The corresponding results for CKFPU are shown in Figure 7c,d. Figure 7e,f are the unwrapped and rewrapped maps based on MCF when the pre-filtering threshold is set at 0.1. Figure 7g,h are the unwrapped and rewrapped maps based on MCF when the pre-filtering threshold is set at the default value 0.25.

From the initial interferograms of Dataset A and Dataset B, it can be seen that the unfiltered interferograms contain high noise content. Pre-filtering must be implemented before the MCF phase unwrapping. However, the unwrapped results based on MCF vary under different filtering thresholds, so it is difficult to select a proper threshold for different interferograms. On the one hand, the exact phase information cannot be unwrapped if there is insufficient pre-filtering. On the other hand, detailed phase information may be lost if there is too much pre-filtering. In contrast, the CKFPU method can get a similar or superior phase unwrapping result without pre-filtering, as shown in Figure 3b,e and c,d. This method not only removes a lot of noise but also achieves a satisfactory unwrapped map. In other words, the CKFPU method can avoid the pre-filtering dilemma to some extent. This is a typical advantage of the CKFPU method. In addition, there is some difference between the results of Datasets 1 and 2. For Dataset 1, the maximum subsidence calculated by CKFPU is much larger than the MCF method. According to the real measurements (introduced in Section 4.1.1 Dataset A), the former is closer to reality. For Dataset 2, the unwrapped results are similar for both CKFPU and MCF. This is most likely due to the fact that the interferogram for Dataset A has dense fringes, which are sensitive to pre-filtering and cause loss of detailed information.

In summary, from the results of these two datasets, the CKFPU method shows similar or even better phase unwrapping performance compared with MCF and it can avoid or relieve the dilemma of parameter setting for pre-filtering to some extent.

#### 4.2.3. Case 3

**Figure 8:**
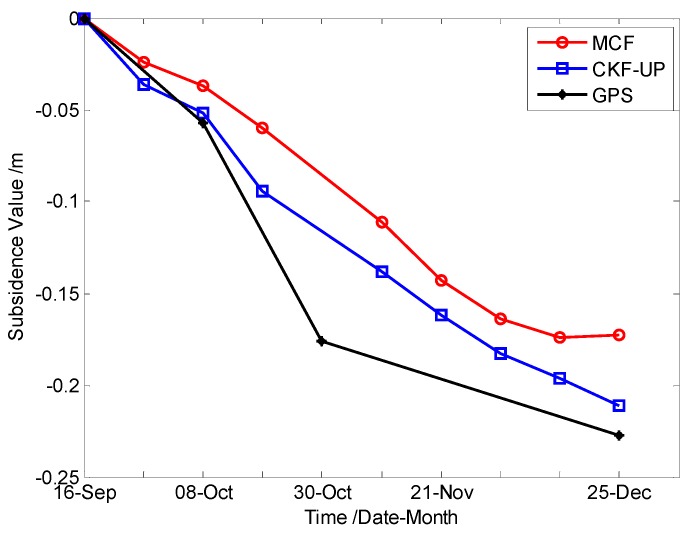
Time series displacement of a Corner Reflector over 88 days, derived from MCF, CKFPU and GPS.

In this case, Dataset 3 was used to demonstrate the performance of CKFPU for areas with high noise. A series of differential interferograms were processed and a Corner Reflector located in the subsidence area was chosen for this comparative analysis. Table 3 and Figure 8 are the numerical and visual results (for conciseness, SSV = Single Subsidence Value and ASV = Accumulated Subsidence Value).

**Table 3:** Results of MCF and CKFPU.

ID	SAR Data		Pre-Filtering + MCF		CKFPU		GPS Result
	Master	Slave	SSV/m	ASV/m	SSV/m	ASV/m	ASV/m
1	16 September 2012	27 September 2012	−0.024	−0.024	−0.036	−0.036	——
2	27 September 2012	8 October 2012	−0.013	−0.037	−0.016	−0.052	−0.057
3	8 October 2012	19 October 2012	−0.023	−0.060	−0.042	−0.094	——
4	19 October 2012	10 November 2012	−0.051	−0.111	−0.044	−0.138	−0.176
5	10 November 2012	21 November 2012	−0.032	−0.143	−0.024	−0.162	——
6	21 November 2012	2 December 2012	−0.020	−0.164	−0.021	−0.183	——
7	2 December 2012	13 December 2012	−0.011	−0.174	−0.013	−0.196	——
8	13 December 2012	24 December 2012	0.002	−0.173	−0.005	−0.201	−0.227

According to GPS data processing of the carrier phase based differential GPS approach, the monitoring point moved −0.227 m in the vertical direction from 16 September 2012 to 24 December 2012 (see Figure 8 and Table 3). The InSAR derived result series (MCF and CKFPU methods) show quite a similar trend with GPS, although the accumulated subsidence are −0.173 and −0.201 m respectively, which are smaller than that observed by GPS. From the collected mine materials and natural environment, the monitoring point location was in a state of slow subsidence when the data was obtained and several differential interferograms contained high levels of noise due to vegetation, dew and snow. Therefore, the interferogram pairs are seriously uncorrelated, which explains the differences between the GPS and InSAR monitoring results. In summary, the proposed CKFPU method can be regarded as a suitable method for phase unwrapping in mining areas with high noise.

## 5. Conclusions

A method based on CKF has been proposed for phase unwrapping in high noise areas, which has implemented phase unwrapping and noise filtering simultaneously. Some important indexes for CKFPU have been analyzed. The results indicate that the number of multi-looks is very important and FD should be considered as the best index for guiding the path of the phase unwrapping. Two case studies indicated the feasibility of CKFPU for DInSAR phase unwrapping areas with high noise and large phase gradients. The performances of CKFPU and MCF were compared. The results indicate that CKFPU is an appropriate phase unwrapping method in mining areas with high noise. In the future, three dimensional phase unwrapping will be further studied to improve the performance of CKFPU.
